# Age of information of a server with energy requirements

**DOI:** 10.7717/peerj-cs.354

**Published:** 2021-03-01

**Authors:** Josu Doncel

**Affiliations:** University of the Basque Country, UPV/EHU, Leioa, Spain

**Keywords:** Age of information, Performance evaluation

## Abstract

We investigate a system with Poisson arrivals to two queues. One queue stores the status updates of the process of interest (or data packets) and the other handles the energy that is required to deliver the updates to the monitor. We consider that the energy is represented by packets of discrete unit. When an update ends service, it is sent to the energy queue and, if the energy queue has one packet, the update is delivered successfully and the energy packet disappears; however, in case the energy queue is empty, the update is lost. Both queues can handle, at most, one packet and the service time of updates is exponentially distributed. Using the Stochastic Hybrid System method, we characterize the average Age of Information of this system. Due to the difficulty of the derived expression, we also explore approximations of the average Age of Information of this system.

## Introduction

### Motivation

The Age of Information has been introduced recently as a metric to measure the freshness of information in applications in which a monitor at the destination is interested in having timely updates about a process of interest in the source. There is a wide range of applications in which the Age of Information is a useful metric of the performance of the system. For example, in autonomous driving, the location, orientation, and speed of the rest of the vehicles in the route must be as recent as possible for an optimal system behavior. Most of the articles that study the Age of Information model the network where the communication network connecting the system whose status is of interest and the monitor that tracks the status of that system as a queueing network. In this work, we analyze the Age of Information of a queueing system with energy requirements, that is, we consider that the communication system needs some energy to deliver the status updates of the source to the monitor.

In the last decades, there has been a big increase of energy usage that come from renewal sources, such as solar or wind and this growth will continue in the near future. This kind of energy sources are known to be very volatile. For instance, the amount of energy generated by solar or wind generators depend on the weather of the region where the generators are located. Therefore, in a system that run on renewal energy, it might happen the generation of energy when it is not required or the lack of energy when the system needs to work. A solution to this problem that has been presented in the last years consists of considering energy harvesting models. In fact, in these models, it is considered that energy can be stored in a battery or in some other device to be used afterwords if this energy is not required at the moment where it is generated. In this work, we consider that the energy is generated according to a random process (which models its generation by renewal sources) and it can be harvested.

### Related Work

The Age of Information metric has been introduced in Kaul et al. (2011) to analyze the freshness of information that a monitor receives from the status updates of a process of interest though a communication channel. More precisely, it is defined as the time elapsed from the generation of the last successfully delivered status update. The authors in Kaul, Yates & Gruteser (2012) consider that the channel by which the status updates are sent to the monitor is a M/M/1 queue, a M/D/1 queue and a D/M/1 queue. The authors in Kam, Kompella & Ephremides (2014) and Kam et al. (2015) consider a channel that consists of a M/M/2 queue. These articles put in evidence that the policies that minimize well-known metrics of queueing theory, such as delay or throughput, are not optimal for the Age of Information. Therefore, since then, there is an active number of researchers that have been interested in analyzing the Age of Information and applying it in a wide range of applications such as content caching Kam et al. (2017a), Yates et al. (2017), Zhong, Yates & Soljanin (2018), ad-hoc networks Kam et al. (2017b), wireless camera networks He, Dan & Fodor (2018, 2019), vehicle -assisted IoT networks Jia et al. (2019), broadcast wireless networks Kadota et al. (2016), Hsu, Modiano & Duan (2017) and Kadota et al. (2018) and the optimal design of freshness-aware IoT Abd-Elmagid, Pappas & Dhillon (2019). The authors in Chaintreau, Le Boudec & Ristanovic (2009) present an age-based model, but in a different context that the Age of Information; in fact, they consider a network with moving nodes of different classes and analyze the effect of gossiping in the occupancy measure of this network

We would like to remark that energy harvesting has been also studied in the context of the Age of Information metric. For instance, the authors in Farazi, Klein & Brown (2018) consider a M/M/1 queue with infinite buffer and last-come-first-served discipline. Another related work is Gindullina, Badia & Gündüz (2020) where the authors study the scheduling that minimizes the average Age of Information in a system with energy harvesting. Our work differs from these ones since we compute an analytical expression of the average Age of Information of a system in a M/M/1/1 queue where the battery is modelled as a M/M/1/1 queue. For a detailed presentation of the recent work about this metric we refer to the following surveys (Sun et al., 2019; Kosta et al., 2017a).

The analytical study of the Age of Information faces a problem to calculate the average Age of Information, one needs to compute the expected value of the product between the random variable of the interarrival times and of the sojourn time, which are dependent random variables. This makes the computation of the average Age of Information a very challenging task for many models. As a result, some authors have been interested in studying other metrics different from the average Age of Information, such that the Peak Age of Information (Costa, Codreanu & Ephremides, 2016), the non-linear age penalty function (Kosta et al., 2017b, 2020) and the age of incorrect information (Maatouk et al., 2020). Recently, the authors in Yates & Kaul (2019) present the Stochastic Hybrid System (SHS) method as a technique to compute the average Age of Information without requiring the computation of the aforementioned difficult expression. We describe this methodology more in detail in “SHS for the Average Age of Information” since it is the technique we use in this article to compute the average Age of Information of our model.

The queueing system we analyze is a particular case of the Energy Packet Network (EPN) model where the queues M/M/1/1 queues. The EPN model was introduced by Gelenbe and his colleagues (Gelenbe, 2011, 2012; Gelenbe & Ceran, 2015) and it is based on G-networks Gelenbe (1991), Gelenbe (1993a, 1993b), Chao, Miyazawa & Pinedo (1999) and Fourneau & Gelenbe (2017) (although not all EPN models are related to G-networks, see for instance, Gelenbe (2014) and Abdelrahman & Gelenbe (2016). Therefore, there is a large number of EPN models where the existence of a product form of the steady-state distribution of jobs in the queues follows from that of G-networks. The importance of the existence of the product form expression is that it allows to optimize the system for some utility function like losses or response time (Gelenbe & Ceran, 2016; Fourneau, Marin & Balsamo, 2016) and the energy distribution (Gelenbe & Zhang, 2019; Zhang, 2019) as well as to design networks (Kadioglu & Gelenbe, 2016; Gelenbe & Abdelrahman, 2018; Doncel & Fourneau, 2019) and data-centers Fourneau (2020) with energy harvesting. We refer to Ray (2019) for a recent survey of the EPN literature. In the EPN model, energy is represented by packets of discrete units called energy packets that model a certain number of Joules, whereas the workload is represented by data packets. Each station of the network consists of a data queue where data packets are stored and an energy queue (or battery) to keep the energy. In such a model, the authors in Gelenbe & Marin (2015) study the influence of the lack of energy in the delay of data packets. As in Kadioglu & Gelenbe (2019), we consider an EPN model where the data packets start that transfer. This means that, when a data packet is served, it is sent to the energy queue and triggers the deletion of an energy packet and the movement of the data packet to the following station, whereas the data packet is lost if it does not find an energy packet. To the best of our knowledge, the average Age of Information of this kind of systems has not been studied so far.

### Contributions

In this article, we study the average Age of Information of a queueing system that is formed by two queues: one queue stores the updates of the system of interest and the other stores the energy that is required to deliver the status updates to the monitor. Both queues can handle, at most, one packet. We assume that there are leakages in the energy queue.

The main contributions of this article are summarized as follows:

- We use the SHS technique to study the average Age of Information of a queueing system under consideration. We first observe that the average Age of Information requires to compute some of the variables that are the solution of a system with 8 equations. Then, we prove that, to compute the average Age of Information, it is enough to solve a system with four equations and four variables. Solving the latter system of equation, we provide an explicit expression of the average Age of Information of our model.
- Due to the difficulty of the derived expression, we aim to study approximations of the average Age of Interest of this model. We first study the accuracy of the expression obtained under the assumption that there is always energy available to deliver the updates and we show that this expression is a good approximation when the arrival rate of energy packets is very large and the rate at which energy packets are lost is small. Then, we present another expression where it provides a good approximation of the average Age of Information when the arrival rate of energy packets is large and any value of the rate at which energy packets are lost.

The results of this article provides insides on the impact on the average Age of Information of the presence of energy harvesting devices in which the arrival of the energy follows a random process. This is a particularly interesting problem given that, in the last decades, there has been a huge growth of energy that come from renewable sources. This work also shows the difficulty in obtaining an analytical expression of the average Age of Information for energy harvesting models and illustrate that the study of approximations of such a difficult performance metric is a very promishing research line.

### Organization of the paper

The remainder of the article is organized as follows. In “Model Description”, we describe the model we study. In “Exact Computation of the Average Age of Information”, we provide an exact analysis of the average Age of Information of our model, whereas in “Approximation of the Average Age of Information” we present approximations to the derived expression. Finally, we explain the main conclusions of our work in “Conclusions”.

## Model Description

### Age of information

We study the transmission of status updates to a monitor. We assume that update *i* is generated at time *s*_i_ and that it is received by the monitor at time $t^{\prime}_i$. Let *N*(*t*) be the index of the last successfully received update at time *t*, that is,

$$N(t)= \max\{i | t_i^\prime\leq t\}.$$

From the above definitions, it follows that the generation time of the last received update at time *t* is given by *t_N(t)_*. The Age of Information is defined as

$$\Delta(t)=t-t_{N(t)}.$$

**Figure 1:**
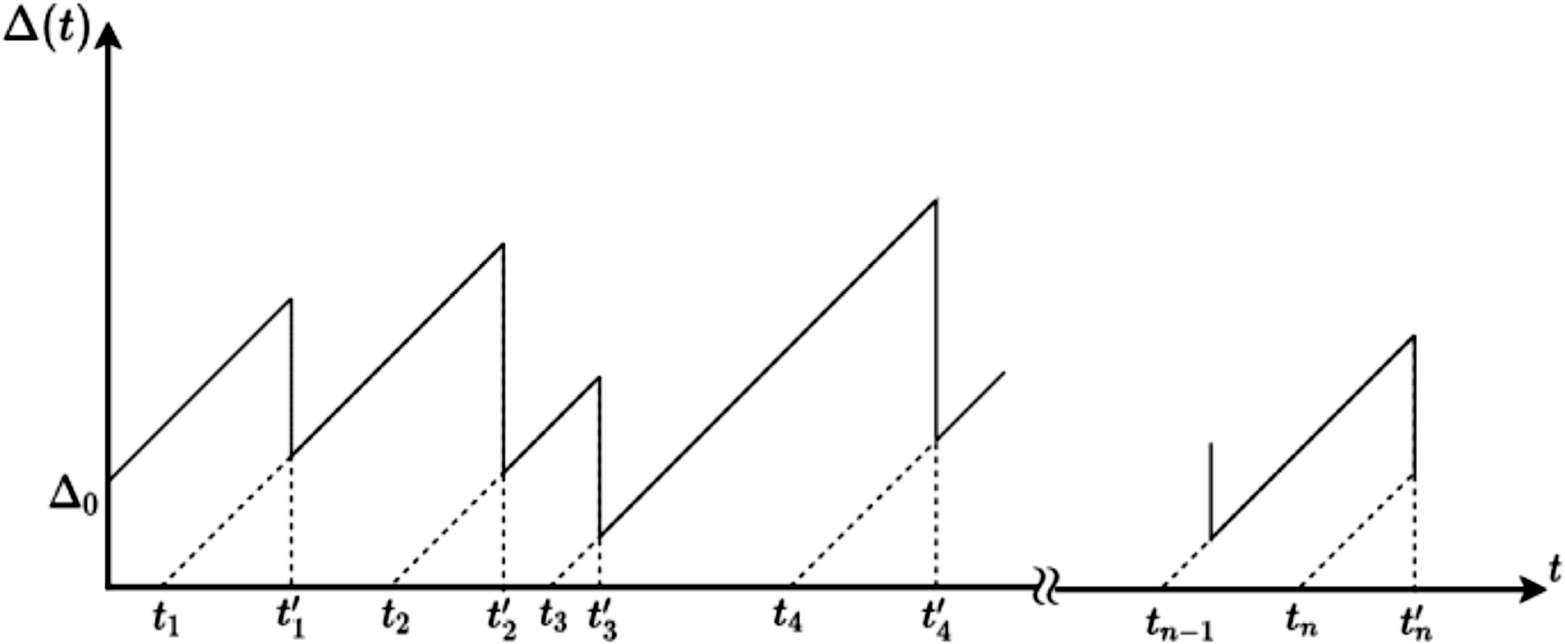
An example of Δ(*t*).

An example of the evolution of Δ(*t*) is presented in Fig. 1.

We know that the average Δ(*t*) exists in a stationary ergodic updating system. Under such assumption, it is defined as follows:

$$\Delta=\lim_{{\rm \tau}\to\infty}\frac{1}{\rm \tau}\int_0^{\rm \tau}\Delta(t)dt.$$

In this article, we are interested in calculating the average Δ(*t*) in an energy harvesting model. As we said in the introduction, the analytical study of the above expression in a general setting is known to be an extremely difficult task. In the following section, we describe for completeness the SHS method and how it can be used to calculate the average Age of Information.

### SHS for the average age of information

We now present the SHS method and how it is used to compute the average Age of Information for an arbitrary model. For full details about this technique, we refer to Hespanha (2006).

In the SHS, the system is modeled as a hybrid state (*q*(*t*), *x*(*t*)). Each of the components of this hybrid state is as follows: *q*(*t*) belongs to a continuous time Markov Chain and *x*(*t*) is a vector evolves according to an ordinary differential equation. The latter contains the current value of the Age of Information as well as the value of the Age of Information if the packets present in the system were delivered to the monitor.

A link *l* of the Markov Chain represents a transition between two states, which occurs with rate λ^l^. In each transition *l*, the vector *x* changes to *x*^*′*^ using a transformation matrix **A**_l_, that is, *x*′ = **x A**_l_. Therefore, **x**(*t*) is a piecewise function.

For each state *q*, we define **b**_q_ as the vector whose elements are zero or one since the time from the generation of the updates that are present in the system increase at unit rate, whereas the updates that are not in the system do not.

We assume the Markov Chain is ergodic and we denote by π_q_ the stationary distribution of state *q*. We denote by ${{\mathcal{L}}_q}$ the set of outgoing links of state *q* and ${\mathcal{L}}^{\prime}_q$ the set of links that get into state *q*. We now present the following theorem that will be used to characterize the average Age of Information:

**Theorem 1** (Yates & Kaul, 2019, Thm 4). *Let v*_q_*(i) denote the i-th element of the vector v*_q_. *For each state q, if v*_q_ *is a non-negative solution of the following system of equations*

(1) $${{\bf v}_q}\sum\limits_{l \in {{\mathcal{L}}_q}} {\lambda ^l} = {{\bf b}_q}{{\rm \pi}_q} + \sum\limits_{l \in {\mathcal{L}}^{\prime}_q} {\lambda ^l}{{\bf v}_{{q_l}}}{{\bf A}_l}$$

then the average Age of Information is $\Delta = \sum\nolimits_q {v_q}(0)$.

In “Exact Computation of the Average Age of Information”, we use the SHS technique to characterize the average Age of Information of a model that consists of single server with energy requirements. Prior to that, we describe this energy harvesting model.

### Energy harvesting model

We analyze a system formed by one server that stores the updates of the status of a system that is monitored. Updates arrive to the system according to a Poisson process with rate λ. We model the server that handles the status updates as a M/M/1/1 queue, that is, it is a server with exponential service times and without buffer. We allow preemption in updates in service, that is, when an update arrives and there is server is busy, the new packet replaces the update that was getting served.

We consider that the updates require energy to be delivered to the monitor. Energy is represented by packets of discrete units called energy packets that model a certain number of Joules. We consider that energy packets are stored in a battery (or energy queue). We assume that energy packets arrive according to a Poisson process with rate rate α. We consider that the battery can store, at most, one energy packet. We also consider that there are energy leakages, that is, an energy packet in the energy queue can be lost with exponential times. We denote by β the leakage rate of one energy packet in the energy queue.

We now present the dynamics of the system. When a status update is served, it is sent to the energy queue and two things can happen: (i) the energy queue is empty, in which can the status update is lost and (ii) there is a packet in the battery, in which case the update is delivered to the monitor and the energy packet disappears.

**Figure 2:**
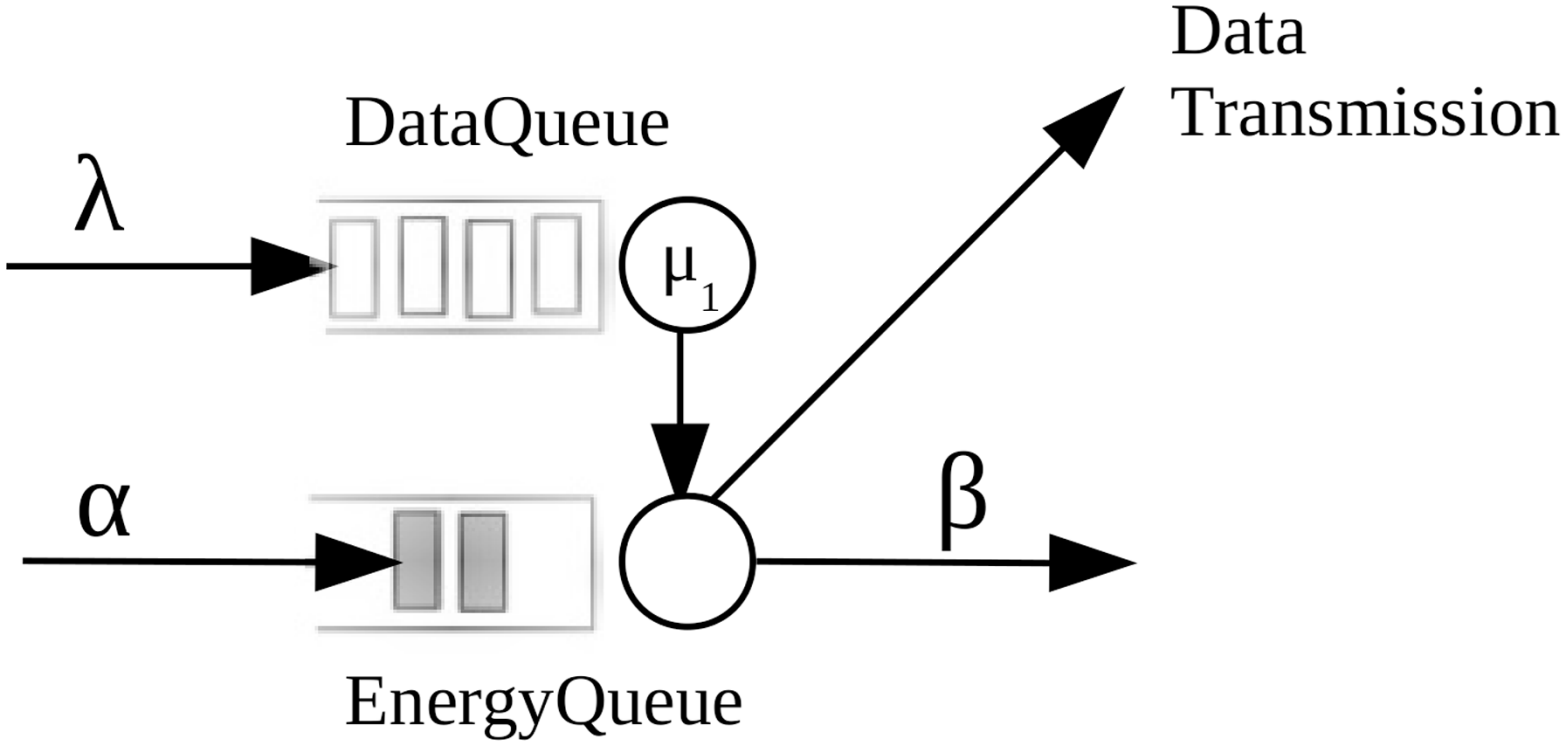
The system model under study.

The model we study in this article is represented in Fig. 2.

## Exact Computation of the Average Age of Information

We use the SHS technique to compute the average age of information of the model described above. The continuous state is **x**(*t*) = [*x*_0_(*t*) *x*_1_(*t*)], where *x*_0_ is the current age and *x*_1_ is the age if the data packet that is getting service is successfully sent to the monitor.

**Figure 3:**
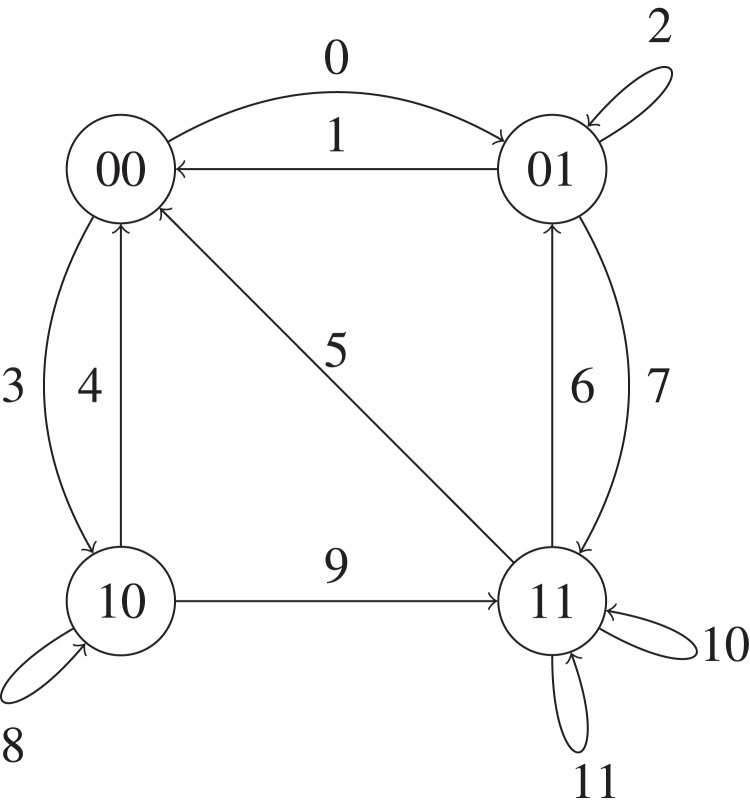
The SHS Markov Chain for the EPN model with a single block.

The discrete state is a continuous time Markov Chain of four states, where the state *ij* represents that there are *i* energy packets and *j* data packets in the system, with *i* = 0,1 and *j* = 0,1. See Fig. 3.

We use the same notation than in Yates & Kaul (2019) and Yates (2018) and we label each of the transitions of the Markov chain with a number from 0 to 11 in Fig. 3. Let $l = 0, \ldots ,11$. We now explain each of the transitions of the Markov chain of Fig. 3 as well as how the state **x** evolves when each of the possible transitions occurs.

*l* = 0 A data packet arrives to the system when the data queue. For this case, this data packet starts getting service and the value of *x*_1_ changes to zero since a new packet arrived, that is, $x^{\prime}_1 = 0$. The value of *x*_0_, however, remains unchanged, $x^{\prime}_0 = {x_0}$. When this transition occurs, the state 00 of the Markov chain of Fig. 3 changes to 01.

*l* = 1 A data packet ends the service and is sent to the energy queue when it is empty. When this occurs, the transfer is unsuccessful and, since the monitor does not receive the data packet, the value of *x*_0_ does not change. When this transition occurs, the state 01 of the Markov chain of Fig. 3 changes to 00.

*l* = 2 A data packet arrives to the system when there is another data packet getting service. Since we allow preemption in service, the freshest data packet starts getting service and the data packet that was previously in service is lost. Therefore, the value of *x*_1_ changes to zero and the value of *x*_0_ does not change. When this transition occurs, the state 01 of the Markov chain of Fig. 3 does not change.

*l* = 3 An energy packet arrives when the system is empty. For this case, the value of *x*_0_ and *x*_1_ are not modified. When this transition occurs, the state 00 of the Markov chain of Fig. 3 changes to 10.

*l* = 4 A leakage of an energy packet occurs and the system gets empty. Besides, the value of *x*_0_ and of *x*_1_ do not change. When this transition occurs, the state 10 of the Markov chain of Fig. 3 changes to 00.

*l* = 5 A data packet finishes the service and is sent to the energy queue, which has an energy packet. Since the transfer is successful, the age at the monitor is updated to the age of the sent data packet, that is, $x_0\prime = {x_1}$. When this transition occurs, the state 11 of the Markov chain of Fig. 3 changes to 00.

*l* = 6 A leakage of an energy packet occurs when the system is full. For this case, the value of *x*_0_ and *x*_1_ are not modified. When this transition occurs, the state 11 of the Markov chain of Fig. 3 changes to 01.

*l* = 7 A data packet arrives to the system when the data queue is empty and the energy queue full. Given that a fresh data packet arrived to the system, the value of *x*_1_ changes as follows: $x_1\prime = 0$. The monitor does not receive packets and, as a result, the value of *x*_0_ is not modified. When this transition occurs, the state 01 of the Markov chain of Fig. 3 changes to 11.

*l* = 8 A data packet arrives when there is another data packet in service. The new packet starts getting service and, therefore, the value of *x*_1_ changes to zero, that is, $x^{\prime}_1 = 0$. However, the value of *x*_0_ is not modified since the monitor does not receive packets. When this transition occurs, the state 10 of the Markov chain of Fig. 3 does not change.

*l* = 9 An energy packet arrives to the system when the energy queue is empty and the data queue is full. When this occurs, the last arrived energy packet is lost and the value of *x*_0_ and of *x*_1_ do not change. When this transition occurs, the state 10 of the Markov chain of Fig. 3 changes to 11.

*l* = 10 An energy packet arrives to the system when the energy queue and the data queue are full. Thus, the energy packet that arrived last is lost and the value of *x*_0_ and of *x*_1_ are not changed. When this transition occurs, the state 11 of the Markov chain of Fig. 3 does not change.

*l* = 11 An energy packet arrives to the system when the energy queue and the data queue are full. For this case, the value of *x*_0_ remains unchanged since the packets are not sent to the monitor, whereas the value of *x*_1_ is set to zero, i.e, $x^{\prime}_1 = 0$. When this transition occurs, the state 11 of the Markov chain of Fig. 3 does not change.

The SHS transitions of this model are represented in Table 1. In this table, we represent in each row the transitions we have explained above. In the second column of the table, we present the origin state and the destination state of the Markov Chain when a transition occurs and in the third column the rate of each transition. In the next column, we show the evolution of the values *x*_0_ and *x*_1_ for each transition and in the last column, the values $\bar v_{ql}A_l$ that are used in Eq. (1). In this table, we can observe how the vector *x* varies for all the transitions described above.

**Table 1:** Table of SHS transitions of Fig. 3.

*l*	*q*_*l*_*→ q*_*l′*_	λ^*l*^	*x*′ = *x A*_*l*_	*V*′_*q1*_ *A*_*l*_
0	00*→* 01	λ	[*x*_0_ 0]	[*v*_00_(0) 0]
1	01*→* 00	μ	[*x*_0_ 0]	[*v*_01_(0) 0]
2	01*→* 01	λ	[*x*_0_ 0]	[*v*_01_(0) 0]
3	00*→* 10	α	[*x*_0_ 0]	[*v*_00_(0) 0]
4	10*→* 00	β	[*x*_0_ 0]	[*v*_10_(0) 0]
5	11*→* 00	μ	[*x*_1_ 0]	[*v*_11_(1) 0]
6	11*→* 01	β	[*x*_0_ *x*_1_]	[*v*_11_(0) *v*_11_(1)]
7	01*→* 11	α	[*x*_0_ *x*_1_]	[*v*_01_(0) *v*_01_(1)]
8	10*→* 10	α	[*x*_0_ 0]	[*v*_10_(0) 0]
9	10*→* 11	λ	[*x*_0_ 0]	[*v*_10_(0) 0]
10	11*→* 11	α	[*x*_0_ 0]	[*v*_11_(0) *v*_11_(1)]
11	11*→* 11	λ	[*x*_0_ 0]	[*v*_11_(0) 0]

Let ${{\mathcal{Q}}^s}$ be the set of states of the Markov chain of Fig. 3. We denote by π_q_ the stationary distribution of state $q \in {{\mathcal{Q}}^s}$. We now study the stationary distribution of the Markov chain of Fig. 3. We write the balance equations below:

$${{\rm \pi} _{00}}({\rm \alpha} + \lambda ) = {{\rm \pi} _{10}}{\rm \beta} + {{\rm \pi} _{01}}{\rm \mu} + {{\rm \pi} _{11}}{\rm \mu}$$

$${{\rm \pi}_{01}}({\rm \mu} + {\rm \alpha}) = {\rm \beta}{{\rm \pi}_{11}} + \lambda {{\rm \pi}_{00}}$$

$${{\rm \pi}_{10}}(\lambda + {\rm \beta}) = {\rm \alpha} {{\rm \pi}_{00}}$$

$${{\rm \pi} _{11}}({\rm \mu} + {\rm \beta}) = {\pi _{10}}\lambda + {{\rm \pi} _{01}}{\rm \alpha}$$

Using the above expressions and also that $\sum\nolimits_{q \in {{\mathcal{Q}}^s}} {{\rm \pi}_q} = 1$, it results that the stationary distribution of the Markov Chain is

(2a) $${{\rm \pi}_{00}} = \displaystyle{{(\lambda + {\rm \beta}){\rm \mu}} \over {({\rm \alpha} + {\rm \beta} + \lambda )(\lambda + {\rm \mu} )}}$$

(2b) $${{\rm \pi}_{01}} = \displaystyle{{\lambda ({\rm \alpha} {\rm \beta} + (\lambda + {\rm \beta})({\rm \beta} + {\rm \pi}))} \over {({\rm \alpha} + {\rm \beta} + \lambda )(\lambda + {\rm \mu})({\rm \alpha} + {\rm \beta} + \lambda )}}$$

(2c) $${{\rm \pi}_{10}} = \displaystyle{{{\rm \alpha}{\rm \mu}} \over {({\rm \alpha} + {\rm \beta} + \lambda )(\lambda + {\rm \mu})}}$$

(2d) $${{\rm \pi}_{11}} = \displaystyle{{{\rm \alpha} \lambda ({\rm \alpha} + {\rm \beta} + \lambda + {\rm \mu})} \over {({\rm \alpha}+ {\rm \beta} + \lambda )(\lambda + {\rm \mu} )({\rm \alpha} + {\rm \beta} + \lambda )}}$$

We now define the vectors **b**_q_ and **v**_q_ for each state $q \in {{\mathcal{Q}}^s}$. First, for the states *q* ∈ {00,10}, we have that **b**_q_ = [1 0] since the age of the monitor increases at unit rate and, since there is not a packet in service, its age do not need to be taken into account. On the other hand, for the states *q* ∈ {01,11}, we have that *b*_q_ = [1 1], since for this case the age of the monitor and of the packet in service increase at unit rate. Besides, we write for all $q \in {{\rm {\cal Q}}^s}$, *v*_q_ = [*v*_q_(0) *v*_q_(1)].

We note that we obtained all we need to apply Theorem 1. Thus, the system of equations given in Eq. (1) is for this case

(3a) $${{\bf v}_{00}}(\lambda + {\rm \alpha}) = [{{\rm \pi}_{00}}\;0] + {\rm \mu}[{v_{01}}(0)\;0] + \mu [{v_{11}}(1)\;0] + {\rm \beta} [{v_{10}}(0)\;0]$$

(3b) $${{\bf v}_{01}}(\lambda + {\rm \mu} + {\rm \alpha}) = [{{\rm \pi} _{01}}\;{{\rm \pi}_{01}}] + \lambda [{v_{00}}(0)\;0] + \lambda [{v_{01}}(0)\;0] + {\rm \beta} [{v_{11}}(0)\;{v_{11}}(1)]$$

(3c) $${{\bf v}_{10}}(\lambda + {\rm \alpha} + {\rm \beta}) = [{{\rm \pi}_{10}}\;0] + {\rm \alpha}[{v_{00}}(0)\;0] + {\rm \alpha} [{v_{10}}(0)\;0]$$

(3d) $$\eqalign{{{\bf v}_{11}}(\lambda + {\rm \mu} + {\rm \alpha} + {\rm \beta} ) = [{{\rm \pi}_{11}}\;{{\rm \pi}_{11}}] + {\rm \alpha} [{v_{01}}(0)\;{v_{01}}(1)] + {\rm \alpha} [{v_{11}}(0)\;{v_{11}}(1)] \cr\quad+ \lambda [{v_{10}}(0)\;0] + \lambda [{v_{11}}(0)\;0]}$$

Taking into account that Eqs. (3a) and (3c) have one irrelevant variable (i.e., the second element of the vector of both sides of the expression is zero), we conclude that the above expression can be rewritten a system of 6 equations. From the above formula, the computation of the average Age of Information requires the values of *v*_00_(0), *v*_10_(0), *v*_01_(0) and *v*_11_(0). In the following result, we show that *v*_00_(0), *v*_10_(0), *v*_01_(0) and *v*_11_(0) is the solution of a system with four equations.

**Lemma 2** *Let* $A = {{{{\rm \pi}_{11}} + \textstyle{{{\rm\alpha}{{\rm\pi}_{01}}} \over {{\rm \lambda} + \rm\mu + \alpha }}} \over {\rm\lambda + \mu + \beta - \textstyle{{\alpha \beta } \over {\lambda + \mu + \alpha }}}}$. *Then, v*_*00*_*(0), v*_*10*_*(0), v*_*01*_*(0) and v*_*11*_*(0) are the solution of*

(4a) $${v_{00}}(\lambda + {\rm \alpha}) = {{\rm \pi} _{00}} + {\rm \mu} A + {\rm \mu} {v_{01}} + {\rm \beta} {v_{10}}$$

(4b) $${v_{01}}({\rm \mu} + {\rm \alpha}) = {{\rm \pi}_{01}} + \lambda {v_{00}} + {\rm \beta} {v_{11}}$$

(4c) $${v_{10}}(\lambda + {\rm \beta}) = {{\rm \pi}_{10}} + {\rm \alpha} {v_{00}}$$

(4d) $${v_{11}}({\rm \mu} + {\rm \beta}) = {{\rm \pi}_{11}} + {\rm \alpha} {v_{01}} + \lambda {v_{10}},$$

where, for all $q \in {{\mathcal{Q}}^s}$, π_q_ is given in Eq. (1).

*Proof*. We first observe that Eq. (1) can be written as the following system of equations:

(5a) $${v_{00}}(0)(\lambda + {\rm \alpha}) = {{\rm \pi}_{00}} + {\rm \mu} {v_{01}}(0) + {\rm \mu} {v_{11}}(1) + {\rm \beta} {v_{10}}(0)$$

(5b) $${v_{01}}(0)({\rm \mu} + {\rm \alpha}) = {{\rm \pi}_{01}} + \lambda {v_{00}}(0) + {\rm \beta} {v_{11}}(0)$$

(5c) $${v_{01}}(1)(\lambda + {\rm \mu} + {\rm \alpha} ) = {{\rm \pi} _{01}} + {\rm \beta} {v_{11}}(1)$$

(5d) $${v_{10}}(0)(\lambda + {\rm \beta}) = {{\rm \pi}_{10}} + {\rm \alpha} {v_{00}}(0)$$

(5e) $${v_{11}}(0)(\lambda + {\rm \mu} + {\rm \beta}) = {{\rm \pi}_{11}} + {\rm \alpha} {v_{01}}(0) + \lambda {v_{10}}(0) + \lambda {v_{11}}(0)$$

(5f) $${v_{11}}(1)(\lambda + {\rm \pi} + {\rm \beta}) = {{\rm \pi}_{11}} + {\rm \alpha} {v_{01}}(1),$$

where π_00_, π_01_, π_10_ and π_11_ are given in Eq. (2). From Eqs. (5c) and (5f), we get that

$$v_{11}(1)=\frac{{\rm \pi}_{11}+\frac{{\rm \alpha}{\rm \pi}_{01}}{\lambda+{\rm \mu}+{\rm \alpha}}}{\lambda+{\rm \mu}+{\rm \beta}-\frac{{\rm \alpha}{\rm \beta}}{\lambda+{\rm \mu}+{\rm \alpha}}}$$

Therefore, the rest of equation can be written as follows:

$${v_{00}}(0)(\lambda + {\rm \alpha}) = {{\rm \pi}_{00}} + {\rm \mu}{v_{01}}(0) + {\rm \mu}\displaystyle{{{{\rm \pi}_{11}} + \displaystyle{{{\rm \alpha}{{\rm \pi}_{01}}} \over {\lambda + {\rm \mu}+ {\rm \alpha}}}} \over {\lambda + {\rm \mu}+ {\rm \beta}- \displaystyle{{{\rm \alpha}{\rm \beta}} \over {\lambda + {\rm \mu}+ {\rm \alpha}}}}} + {\rm \beta}{v_{10}}(0)$$

$${v_{01}}(0)({\rm \mu}+ {\rm \alpha}) = {{\rm \pi}_{01}} + \lambda {v_{00}}(0) + {\rm \beta}{v_{11}}(0)$$

$${v_{10}}(0)(\lambda + {\rm \beta}) = {{\rm \pi}_{10}} + {\rm \alpha}{v_{00}}(0)$$

$${v_{11}}(0)({\rm \mu}+ {\rm \beta}) = {{\rm \pi}_{11}} + {\rm \alpha}{v_{01}}(0) + \lambda {v_{10}}(0)$$

If we denote $A = \textstyle{{{{\rm \pi}_{11}} + \textstyle{{{\rm \alpha}{{\rm \pi}_{01}}} \over {\lambda + {\rm \mu}+ {\rm \alpha}}}} \over {\lambda + {\rm \mu}+ {\rm \beta}- \textstyle{{{\rm \alpha}{\rm \beta}} \over {\lambda + {\rm \mu}+ {\rm \alpha}}}}}$, the desired result follows.

We now focus the solution of Eq. (2). Since *v*_10_(λ + β) = π_10_ + α *v*_00_, we can restrict to study the following system of three equations:

$${v_{00}}(\lambda + {\rm \alpha}) = {{\rm \pi}_{00}} + {\rm \mu}A + {\rm \mu}{v_{01}} + \displaystyle{{{\rm \beta}{{\rm \pi}_{10}}} \over {\lambda + {\rm \beta}}} + \displaystyle{{{\rm \beta}{\rm \alpha}} \over {\lambda + {\rm \beta}}}{v_{00}}$$

$${v_{01}}({\rm \mu}+ {\rm \alpha}) = {{\rm \pi}_{01}} + \lambda {v_{00}} + {\rm \beta}{v_{11}}$$

$${v_{11}}({\rm \mu}+ {\rm \beta}) = {{\rm \pi}_{11}} + {\rm \alpha}{v_{01}} + \displaystyle{{\lambda {{\rm \pi}_{10}}} \over {\lambda + {\rm \beta}}} + \displaystyle{{\lambda {\rm \alpha}} \over {\lambda + {\rm \beta}}}{v_{00}},$$

where *A* is defined in Lemma 2.

Let

$$M = \left( {\matrix{ {{m_{11}}} & { - {\rm \mu}} & 0 \cr { - \lambda } & {{\rm \mu}+ {\rm \alpha}} & { - {\rm \beta}} \cr { - {m_{31}}} & { - {\rm \alpha}} & {{\rm \mu}+ {\rm \beta}} \cr } } \right),$$

where

(6) $${m_{11}} = \lambda + {\rm \alpha}- \displaystyle{{{\rm \alpha}{\rm \beta}} \over {\lambda + {\rm \beta}}}{\rm and}\;{m_{31}} = \displaystyle{{{\rm \alpha}\lambda } \over {\lambda + {\rm \beta}}}$$

The system of equations we need to solve is the following:

(7) $$M \cdot \left( {\matrix{ {{v_{00}}(0)} \cr {{v_{01}}(0)} \cr {{v_{11}}(0)} \cr } } \right) = \left( {\matrix{ {{b_0}} \cr {{{\rm \pi}_{01}}} \cr {{b_3}} \cr } } \right)$$

where

(8) $${b_0} = {{\rm \pi}_{00}} + {\rm \mu}A + \displaystyle{{{\rm \beta}{{\rm \pi}_{10}}} \over {\lambda + {\rm \beta}}}{\rm and}\;{b_3} = {{\rm \pi}_{11}} + \displaystyle{{\lambda {{\rm \pi}_{10}}} \over {\lambda + {\rm \beta}}}$$

Calculating the inverse of *M*, we obtain the solution of this system and we presented it here.

**Lemma 3** *The solution of Eq. (7) is given by*

$${v_{00}}(0) = \displaystyle{{\rm \mu}\over {det(M)}}({b_0}({\rm \alpha}+ {\rm \beta}+ {\rm \mu}) + {{\rm \pi}_{11}}({\rm \beta}+ {\rm \mu}) + {b_3}{\rm \beta})$$

$${v_{01}}(0) = \displaystyle{1 \over {det(M)}}({b_0}({\rm \beta}{m_{31}} + {\rm \beta}\lambda + \lambda {\rm \mu}) - {{\rm \pi}_{11}}{m_{11}}({\rm \beta}+ {\rm \mu}) - {b_3}{\rm \beta}{m_{11}})$$

$${v_{11}}(0) = \displaystyle{1 \over {det(M)}}({b_0}({\rm \alpha}{m_{31}} + {\rm \alpha}\lambda + {m_{31}}{\rm \mu}) - {{\rm \pi}_{11}}({\rm \beta}{m_{11}} + {m_{31}}{\rm \mu}) - {{\rm \beta}_3}({\rm \alpha}{m_{11}} + {m_{11}}{\rm \mu}+ \lambda {\rm \mu}))$$

where *b*_0_ and *b*_3_ are given in Eq. (8), *m*_11_ and *m*_31_ in (6) and, for all $q \in {{\mathcal{Q}}^s}$, π_q_ is given in Eq. (2).

From Theorem 1, we know that the average Age of Information of this model is given by *v*_00_(0) + *v*_01_(0) + *v*_10_(0) + *v*_11_(0). In the following result, we present its value:

**Proposition 4** *The average Age of Information of a single server with energy harvesting is*

$$\Delta = {v_{00}}(0)\left( {\displaystyle{{\rm \alpha}\over {\lambda + {\rm \beta}}} + 1} \right) + \displaystyle{{{{\rm \pi}_{10}}} \over {\lambda + {\rm \beta}}} + {v_{01}}(0) + {v_{11}}(0),$$

where *v*_00_(0),*v*_01_(0) and *v*_11_(0) are given in Lemma 3.

*Proof*. Using that ${v_{10}}(0)(\lambda + {\rm \beta}) = {{\rm \pi}_{10}} + {\rm \alpha}{v_{00}}(0)$ and the results of Lemma 3, we derive the desired result. □

### The case α→∞

In this section, we consider that the arrival rate of energy packets tends to infinity and the rest of the parameters are finite. We first note that, when α→∞, π_00_ and π_01_ tend to 0, whereas π_10_ and π_11_ tends, respectively, to $\textstyle{{\rm \mu}\over {\lambda + {\rm \mu}}}$ and $\textstyle{\lambda \over {\lambda + {\rm \mu}}}$. On the other hand, from the first three equations of Eq. (1), it follows that *v*_00_(0), *v*_01_(0) and *v*_01_(1) tend to zero when α→∞. Therefore, to compute the Age of Information in this regime we have to analyze the following system of equations:

$${v_{10}}(0)(\lambda + {\rm \beta}) = {{\rm \pi}_{10}} + {\rm \alpha}{v_{00}}(0)$$

$${v_{11}}(0)(\lambda + {\rm \mu}+ {\rm \beta}) = {{\rm \pi}_{11}} + {\rm \alpha}{v_{01}}(0) + \lambda {v_{01}}(0) + \lambda {v_{11}}(0)$$

$${v_{11}}(1)(\lambda + {\rm \mu}+ {\rm \beta}) = {{\rm \pi}_{11}} + {\rm \alpha}{v_{01}}(1)$$

We now study *α v*_00_(0) when α→*∞*. Using L’Hopital’s rule, we get that

$$\lim_{{\rm \alpha}\to\infty}{\rm \alpha}v_{00}(0)={\rm \mu}v_{11}(1)+{\rm \beta}v_{10}(0)$$

Likewise, we show that α *v*_00_(1) tends to β *v*_01_(0) and α *v*_01_(1) tends to β *v*_11_(1) when α→∞. As a result, the above system of equations can be written as follows:

(9a) $${v_{10}}(0)\lambda = {{\rm \pi}_{10}} + {\rm \mu}{v_{11}}(1)$$

(9b) $${v_{11}}(0)(\lambda + {\rm \mu}) = {{\rm \pi}_{11}} + \lambda {v_{01}}(0) + \lambda {v_{11}}(0)$$

(9c) $${v_{11}}(1)(\lambda + {\rm \mu}) = {{\rm \pi}_{11}}$$

From this expression, we derive the following interesting conclusions. First, we observe that this system of equation does not depend on β. This means that the Age of Information of the EPN model with a single station, when *α→∞*, does not depend on the leakage rate of energy packets. Another conclusion is related to the comparison of the above expression with the model studied in Section IVB of Yates & Kaul (2019). We observe that, if we consider λ_2_ = 0 in their model, that is, that there is a single source, both models coincide. More precisely, if we consider *λ*_2_ = 0 in (48) of Yates & Kaul (2019), we obtain the same system of equation as the one above. Therefore, the average Age of Information of a single station when α→∞ is the same as that of a single server with preemption in service without energy hasvesting and its value can be derived from Theorem 2 of Yates & Kaul (2019).

**Proposition 5** *The average Age of Information of a single server with energy harvesting when α tends to infinity is*

$$\Delta=\frac{1}{\rm \mu}+\frac{1}{\lambda}$$

*Proof*. See the proof of Theorem 2 of Yates & Kaul (2019).

## Approximation of the Average Age of Information

In Proposition 4, we characterize the average Age of Information of a single server with energy harvesting. Due to the difficulty of the derived expression, we aim to study approximations of the average Age of Information in this section.

We have shown in Proposition 5 that, when α→∞ and the rest of the parameters are finite, the average Age of Information of a single server with energy harvesting tends to

$\displaystyle{1 \over {\rm \mu}} + \displaystyle{1 \over \lambda }$       (AOI-APPROX)

**Figure 4:**
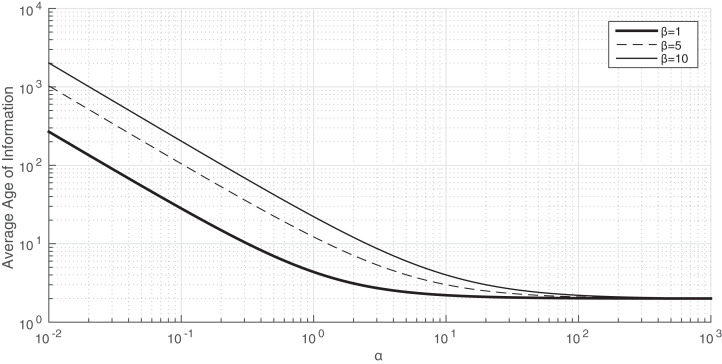
The average Age of Information of Proposition 4 when a varies from 0.01 to 10^3^. *x*-axis and *y*-axis in logarithmic scale. λ = μ = 1.

Therefore, this expression can be used as an approximation of the average Age of Information provided in Proposition 4 when α is large. We first study the accuracy of this approximation in Fig. 4.

We consider λ = μ = 1 and we plot in Fig. 4 the evolution of the average Age of Information using the result of Proposition 4 when α changes from 0.01 to 10^3^ and different values of β. We observe that, in all the cases, when α is large, the average Age of Information is very close to 2, which is the value obtained for the approximation (AOI-APPROX), that is, for $\textstyle{1 \over {\rm \mu}} + \textstyle{1 \over \lambda }$. Interestingly, for β = 1, the approximation is very accurate when α is larger than 10. However, for β = 5 and β = 10, the approximation is accurate when α is larger than 100. Therefore, from this experiment we conclude that the range of values of α such that the accuracy is good decreases with β.

Another interesting conclusion of the experiments represented in Fig. 4 is that the average Age of Information is very large when α is small. For instance, when β = 1 and α = 10^−2^, the average Age of Information is 2· 10^3^. The raison for this is that, when the arrival rate of energy packet is very small, the energy queue is almost always empty. Thus, when a data packet is sent to the energy queue, it is almost always lost and, therefore, since the monitor does not receive timely status updates, the average Age of Information is large.

**Figure 5:**
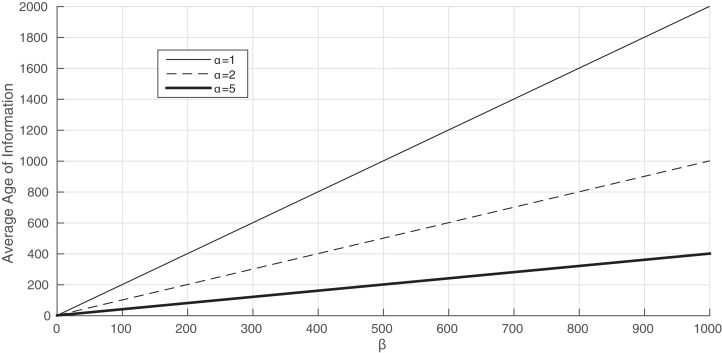
The average Age of Information of Proposition 4 when b varies from 0 to 10^3^. λ = μ = 1.

We now analyze the influence of the leakage rate on the average Age of Information. In Fig. 5, we consider λ = μ = 1 and different values of α. We first see in this plot that, when the leakage rate is large, the average Age of Information is high. For instance, when α = 1 and β = 100, the average Age of Information equals 2,000. Another interesting property we derive from this illustration is related to the monotonicity of the average Age of Information with β. In fact, we show that the average Age of Information increases linearly with β. Therefore, this illustration shows the existence of an approximation of the average Age of Information that depends linearly on the leakage rate. Moreover, the average Age of Information increases faster for smaller values of the arrival rate of energy packets.

Following the conclusions obtained from Fig. 5, we propose the following approximation of the average Age of Information for the model of a single server with energy harvesting:

$\displaystyle{1 \over \lambda } + \displaystyle{1 \over {\rm \mu}} + \displaystyle{{2{\rm \beta}} \over {\rm \alpha}}$       (REFINED-AOI-APPROX)

As it can be seen it is a refined version of the approximation of (AOI-APPROX) since it has an additional term $\textstyle{{2{\rm \beta}} \over {\rm \alpha}}$. We will see that this additional term leads to a high improvement of the accuracy when β is large.

**Figure 6:**
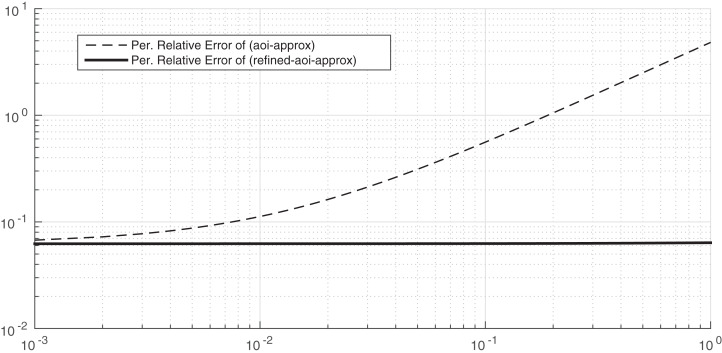
Percentage relative error comparison of the approximations (AOI-APPROX) and (REFINED-AOI-APPROX) over β. *x*-axis and *y*-axis in logarithmic scale. λ = μ = 1 and α = 20.

In Fig. 6, we consider λ = μ = 1 and α = 20. As we before, the approximation (AOI-APPROX) provides approximates the average Age of Information. We now aim to compare the accuracy of the approximation given in (REFINED-AOI-APPROX) with the accuracy of the approximation of (AOI-APPROX) when β varies from 0.001 to 1. As it can be observed, the accuracy of both approximations is very high for small values of the leakage rate since the percentage relative error in both cases is almost 5·10^−2^%. However, the percentage relative error of the approximation REFINED-AOI-APPROX does not substantially change with β, whereas for the approximation (AOI-APPROX) it increases substantially; indeed, when β is equal to one, the percentage relative error of the approximation (AOI-APPROX) is 4%. Therefore, from this plot we conclude that, when α is high and β small, both approximation are accurate, but when β and α are high, the approximation (REFINED-AOI-APPROX) is much more accurate than the approximation (AOI-APPROX).

**Figure 7:**
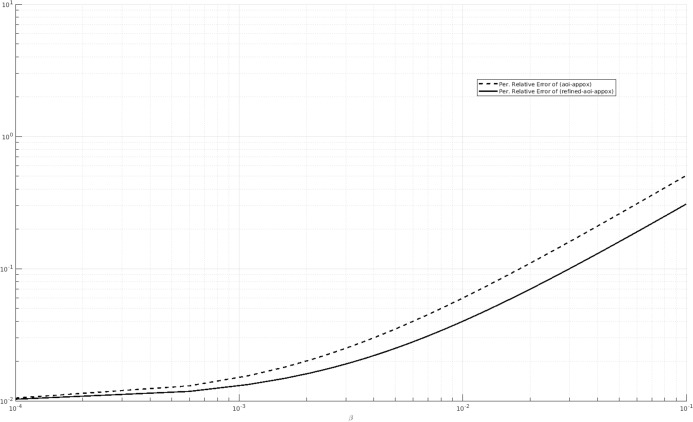
Percentage relative error comparison of the approximations (AOI-APPROX) and (REFINED-AOI-APPROX) over β. *x*-axis and *y*-axis in logarithmic scale. λ = 1, μ = 0.25 and α = 20.

**Figure 8:**
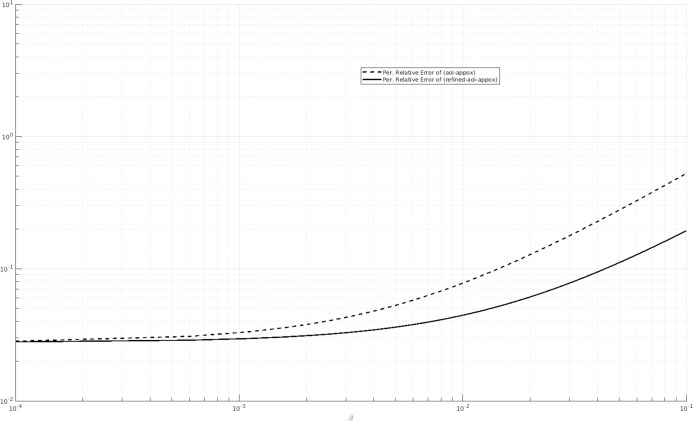
Percentage relative error comparison of the approximations (AOI-APPROX) and (REFINED-AOI-APPROX) over β. *x*-axis and *y*-axis in logarithmic scale. λ = 0.5, μ = 1 and α = 20.

We also consider other values of the parameters and we compare the percentage relative error of (AOI-APPROX) and (REFINED-AOI-APPROX) when β varies. For instance, in Fig. 7, we consider λ = 1, μ = 0.25 and α = 20 and in Fig. 8, we consider λ = 0.5, μ = 1 and α = 20. We observe that, in both cases, the percentage relative error of (AOI-APPROX) is larger that that of (REFINED-AOI-APPROX).

## Conclusions

We analyze the average Age of Information in a system with two queues, one queue that stores the updates and another queue that handles the energy. We consider that an update is sent to the energy queue when it ends the service and, if there is an available energy packet, the status update is transferred to the monitor (and the energy packet leaves the system), whereas if the energy queue is empty, the update is lost. We consider that energy packets and updates arrive to the system according to independent Poisson processes. We characterize the average Age of Information of this system using the SHS technique and we show that its value coincides with the sum of the variables of a system of 4 equations. Given the difficulty of the derived expression, we also provide different approximations of the average Age of Information of this system. We believe that the obtained results are useful in the design of systems with energy requirements since the derived expression (and the approximations) allows us to formulate optimization problems related to the energy consumption of systems where the freshness of information is the performance metric of interest.

For future work, we are interested in studying the EPNs models from the perspective of the the Age of Information metric. In fact, as said in the Related Work Section, the EPN models have the nice property of the existence of a product form expression of the steady-state distribution of packets. We believe that this property can provide fruitful results in the analysis of the average Age of Information for general EPN models.

## Supplemental Information

10.7717/peerj-cs.354/supp-1 Supplemental Information 1 Code to generate Figure 4.

10.7717/peerj-cs.354/supp-2 Supplemental Information 2 Code to generate Figure 5.

10.7717/peerj-cs.354/supp-3 Supplemental Information 3 Code to generate Figure 6.

## Additional Information and Declarations

### Data Availability

The following information was supplied regarding data availability: MATLAB code is available in the Supplemental Files.
